# Obituary Notice of the Late William Power, M. D., of Baltimore

**Published:** 1853-05

**Authors:** 


*Obituary Notice of the late William Power, M. D., of Baltimore.*—The following eloquent tribute to the memory of the late Dr. Power, is contained in the April No. of the American Journal of the Medical Sciences. The initials show it to be from the pen of Dr. Alfred Stille, of Philadelphia. It is written in the chaste, elegant style for which Dr. S. is distinguished. But its merit, and the interest with which it will be read, involve something higher than merely literary excellence. It inspires admiration for the character of the deceased, whose example is so worthy of imitation, and, at the same time, a profound respect for the qualities of the heart which have prompted so touching an eulogy:

Died, in Baltimore, on the 15th of August, 1852, and in the 39th year of his age, William Power, M. D., late Professor of the Theory and Practice of Medicine in the University of Maryland.

When the young die, we grieve for the hopes that have faded like the flowers that wither on their bier; when the places of the old know them no more forever, we mourn that the tree which had long sheltered us, and been perchance a landmark to the wandering, has fallen from its station; but when death smites the vine that was still giving us nourishment with its fruit and refreshment with its shade, how sadly tender is the memory of its fragrant blossoms, how mournful and desolate seems the future of whose riches it had offered so liberal a pledge. Such was our departed friend. His nature, his culture, his earlier, and his maturer fruits, combined to make his life a blessing to those who partook of his gifts or dwelt within his shadow; but, even whilst supplying others most largely, a worm was at his own core; so one by one his branches withered and he fell away from among the living.

His life was a life of earnest study, and of noble ambition. He came into the world of professional action thoroughly furnished with all that that could make him a wise and accomplished physician. Just twenty years before his death he took the degree of A. B. at Yale College, and in the following year commenced in the office of Dr. Buckler, a course of study, which, until the last year of his life, he never ceased zealously to pursue. In 1834, he entered, as a Resident Student, the Baltimore Almshouse Hospital, the institution of which he was destined to become, in later years, the most distinguished ornament. After graduating in medicine, and performing his hospital duties with ability and zeal, he determined to increase his knowledge by a course of study and observation in Europe, a step of which at that time there were but few examples in his native city.

The writer of this sketch first knew him in the midst of the scientific turmoil of which Paris was then the seat, and can testify to the ardent and steadfast enthusiasm which animated him; to his constant and unwearied attendance at the hospitals, the laborious records of his observations, his intelligent and discriminating discussions of the daily lessons which, in common with a crowd of American listeners, he was learning from the lips of Louis and Chomel, of Andrel and Rostan, of Grisolle and Barth. In the rigid school of the Medical Society of Observation, and of its illustrious president, the astute and penetrating genius of Dr. Power found a severe but salutary discipline, and he always referred to the evenings spent in its exercises as among the most delightful and profitable of his professional life. Its training developed his capacity for the systematic study of disease, and fitted him to become an example and a teacher to the medical students of his native city.

Enthusiastic in his pursuit of knowledge, he immediately, on his return home, sought and obtained anew the post of Resident Physician in the Almshouse Hospital, and only relinquished it when, after nine months' service, he was honored with the appointment of Visiting Physician to the same institution. As a consequence of the disgraceful custom of allowing political partizanship to interfere with the administration of public charities, Dr. Power was removed from his office on a change in the direction of the almshouse; but subsequently, in 1841, was reinstated. In this and in the following year he delivered two courses of lectures on the Physical Exploration of the Chest, at the Baltimore Infirmary, and under the auspices of the Faculty of the University. They were well attended, and gave him considerable reputation for attainments and skill in lecturing. His health now became precarious, and he felt obliged to quit his post, both as clinical teacher and lecturer, nor did he resume its duties until 1844. In the following year, he was appointed Professor of the Theory and Practice of Physic, in the University of Maryland.

The influence of Dr. Power over the young men, who, as students or as assistant physicians, followed his daily instructions, was unbounded. One and all caught somewhat of the enthusiasm which inspired their teacher and stayed him against the crushing weight of his disease. Before his time, we are assured, it was difficult to find candidates for the place of Resident Student in the Hospital; but owing to the interest with which he invested the duties of the office, and the reputation which the institution thereby acquired as a school of medicine, it was now only by an application of a year or more that candidates could secure their election. Even at this period, the physical sufferings of Dr. Power were such that he could hardly go up stairs without panting; yet his love of knowledge and his devotion to duty made him cling resolutely to his post, and point the way to others, although he did not hope long to tread in it himself. It is a sad, and at the same time a singular fact, that of the eight Resident Students who, in 1844–45, were his assistants, all but one preceded him to the grave! How painful a commentary is this on the deadly influences of our professional pursuits on life and health.

The career of Dr. Power, as professor, was a distinguished one. That it was singularly successful is proved by the large number of students whom he drew to the University; by the position he at once assumed among his professional brethren, and by the reputation he soon acquired as a clinical teacher in the College Infirmary. His thorough, systematic, and intelligent study of disease, his accurate diagnosis, and his mode of illustrating one case by another, interested his audience deeply, and accustomed them to observe for themselves. In a word, his whole system of analytical study, as he had learned it from the ablest teachers of Europe, attracted and delighted all of the maturer and best-furnished minds among his hearers, and gradually raised up a class of ardent cultivators of medical science, which has already given to the world some of the fruits of his instruction, and some earnest of their own future attainments. As a lecturer, we are informed, he did not aim at originality, but rested satisfied if what he taught, from whatever source obtained, was *true*. The confidence he inspired was boundless; for did he make a wrong diagnosis, or misstate a fact, he was the first to admit it when discovered, and to confess his error. His lectures were less remarkable for excellence of style and originality of thought, than for their presenting a calm and unbiased arrangement of the opinions of others, corrected and modified by his own experience. In this, as in all else, he held truth to be more valuable than ingenuity, and a positive result than the most elaborate and cunningly devised fiction. Yet, in dealing with the errors and follies of medical systems, he was careful not to forget the charity which is due to the weakness of human nature, nor the distinction, too often lost sight of, between the doctrines he combated and the persons who propounded or adopted them.

Dr. Power's health had never been robust. Emphysema of the lungs, and a consequent derangement of the heart's action, had, for a long time, made active exertion fatiguing to him. As long ago as 1837, a pedestrian tour in Switzerland cost him several paroxysms of severe dyspnœa and palpitation of the heart with hæmoptysis; but he did not become seriously alarmed about his situation until nearly six years afterward. In the autumn of 1843, he was obliged to seek relaxation in Cuba, whence he returned in the following spring, with his health materially improved. He continued better, in the main, although far from being vigorous, and from time to time he showed the effects of too close an application to his duties as hospital physician. His marriage in 1847, and his appointment in the University, which he had received two years before, exerted, doubtless, a very favorable influence upon his health, for he continued to perform with animation and a good degree of vigor, his professiorial duties, to draw around him a crowd of earnest and attached pupils, and in the social circle to win by his amenity, and interest by his varied talents, a large number of sincere and valuable friends. But the disease which had so long been baffled by skill and sedulous attention, at length gave signs that it was obtaining the mastery. During the winter of 1851–2, Dr. P. was unable to perform his public duties, and, in February of the latter year, resigned his professorship. In the month of May, the writer of this notice had the satisfaction of an interview with his old companion. He was wasted and wan indeed, for consumption had nearly worn away his earthly dwelling; but his soul shone all the clearer, and brighter, and purer for its freedom from the clogs of flesh. One might well have expected to hear the devotee of science, and the ambitious teacher, mourn over the unfinished career he had run, and resent the defeat of his wishes. But his spirit had been chastened. Contented with his share of worldly success, without a syllable of complaint at the disappointment of his hopes, he could still take interest in the science which he loved, and find in conversation upon its progress a consolation for his own withdrawal from a share in its rewards. Resigned and cheerful, he awaited with patience, yet with strong desire, the moment of his departure; for, while he longed for a release from suffering, he did not disdain the world in which he had been permitted to fit himself for a better.

For three months after this interview, he waxed feebler and feebler, but his soul was more and more brightened by a light from beyond the grave, and displayed to the last moment the calm hopefulness of one whose heart is fixed on Heaven.

As a student, Dr. Power was intelligent and industrious; as an instructor, he was faithful in duty, thorough in teaching, earnest in manner, clear in exposition, copious in illustration, convincing in argument; as a friend, he was sincere and constant; and, as a man, noble in his aims, pure in his means, a scorner of the false and hollow, and a lover of the real and the true. Thus, true to Heaven and to human duty, he lived respected even by those who did not share his love, and died as they should die who see beyond the grave an immortality of knowledge and of blessedness. A. S.

